# Art–science multidisciplinary collaborations to address the scientific challenges posed by COVID-19

**DOI:** 10.1080/07853890.2022.2123557

**Published:** 2022-09-16

**Authors:** José de la Fuente

**Affiliations:** a Instituto de Investigación en Recursos Cinegéticos IREC-CSIC-UCLM-JCCM, SaBio, Ciudad Real, Spain; b Department of Veterinary Pathobiology, Center for Veterinary Health Sciences, Oklahoma State University, Stillwater, OK, USA

**Keywords:** Protean art, art-based method, mixed-methods, multidisciplinary, COVID

## Abstract

The ongoing coronavirus pandemic COVID-19 constitutes a scientific and social challenge. The application of mixed-methods research with multidisciplinary collaborations increases the success of experimental design and interpretation of results to approach scientific challenges. The objective is to develop and implement protean art algorithms with interactions between artists and scientists for scientific research in areas of molecular biology, immunology, ecology and biomedicine. In this perspective, artists were invited to contribute pieces related to the pandemic, and scientists were then challenged to contribute their view and proposed research inspired by artist contribution to face COVID-19 scientific challenges. Proposed research objectives inspired by artist contributions contribute to approach COVID-19 scientific and social challenges with results that may translate into new diagnosis and control interventions. The proposed research objectives approach vaccine protective mechanisms and the development of nutritional interventions with possible impact on boosting protective response to vaccination, the impact of fuel pollutants on host immunity and virus transmission, the possible role of ectoparasite vectors in the appearance of SARS-CoV-2 variants and virus transmission, collaboration between different sectors to contribute to virus surveillance and reduce risks of contagion, characterization of the incidence of zoonotic diseases during and after the COVID-19 pandemic in relation to modifications in the interactions between humans and reservoir animal species, evaluation of the risks associated with sexual or congenital transmission of SARS-CoV-2, development of new methods for the easy and rapid detection of very low SARS-CoV-2 virus amounts in infected but asymptomatic individuals, and understanding society perceptions about the socio-ecological relationships between decoupled environments and the risks and effects of pandemics. This approach may be used to promote social participation in science through combined scientific and artistic perspectives with impact on science and society.

**KEY MESSAGE**
- Mixed-methods research with multidisciplinary collaborations increases the success of experimental design and interpretation of results.
- Implementation of protean art algorithms through interactions between artists and scientists advances scientific research.
- Proposed research objectives inspired by artist contributions contribute to approach COVID-19 scientific and social challenges with results that may translate into new diagnosis and control interventions.

## Introduction: Basis of the perspective study

Mixed-methods research including the collaboration between art and science increases the success of experimental design and interpretation of results to approach scientific challenges such as those posed by the SARS-CoV-2 coronavirus disease 2019 (COVID-19) [1–7]. As previously discussed [7,8], science inspires artists and art inspires scientists by promoting the exploration of new questions with novel methodological approaches [5,9–12]. Although art may have biological basis [13,14], art stimulates the power of curiosity that scientists frequently lost during research. Artistic and musical representations could translate into complex or unrecognized concepts and provide a way to better understand and approach scientific challenges [3,15]. The collaboration between science and art has been used for communication in different areas and science benefits when artists get involved in research with effective accomplishment of both epistemic and practical research objectives [16]. The objective is to develop and implement protean art algorithms with multidisciplinary collaborations in scientific research with possible impact on molecular biology, immunology and biomedicine [1,7,17–19].

In this perspective, the collaboration between artists and scientists was used to face challenges posed by the COVID-19. Although vaccines have been a major achievement for the control of COVID-19, several questions remain to better understand SARS-CoV-2 transmission and disease symptoms in vaccinated individuals (e.g. Lauring et al. [20]) that raise public concerns (e.g. de la Fuente et al. [21]).

The artists provided their perspective on COVID-19. Then, scientists from different countries/institutions and with research expertise in different disciplines were invited to contribute their views and proposed research inspired by artist perspective in connection to COVID-19. Both artist’s and scientist’s views on the pandemic may be affected by multiple factors such as country of residence and personal experiences.

## Discussion

### Methodological approach

- The study was conducted between March and July 2022.
- Visual artists living in different countries were contacted and invited to participate in the study. The artists were randomly selected based on previous knowledge of their multi style work by the author. The message included a brief description from de la Fuente [1], “Science requires innovative and reasoning strategies to achieve epistemic goals based on empirical evidence to accept or withdraw a hypothesis, and practical goals focussed on research social impact. In scientific strategies, mixed-methods research reduces the risks associated with experimental design and interpretation of results. In this context, the collaboration between art and science provides mixed-methods contributing to innovative approaches to face scientific and social challenges”.
- All the contacted artists accepted to participate and were then asked to contribute images of one to three art pieces related to the COVID-19 with corresponding title, technique, dimensions and year of execution (Table 1). In addition, they were requested to provide their brief description of the message/vision transmitted by the pieces in connection with the pandemic. All artists authorized the publication of the high-resolution images of the pieces and information provided by them.
- Then, scientists working in different countries/institutions and research areas were invited to participate in the study (Table 1). The scientists were randomly selected from author’s collaborators with different areas of expertise including ecology, veterinary medicine, molecular biology, parasitology, biochemistry, biomedicine and physics (for additional information see ORCID records in Table 1).
- All the contacted scientists accepted to participate. Then, one artist proposal with pieces and comments was randomly assigned to them and requested to contribute their view and proposed research inspired by artist contribution to face COVID-19 scientific challenges. Scientists were not in contact with the artists. The scientists raised questions both within and outside their research areas to explore fresh, bold new ideas.
- Proposed research inspired by artist contributions was then used in the arrived order by the author to draft objectives to approach COVID-19 scientific and social challenges with results that may translate into diagnosis and control interventions with publications in scientific journals.
- Finally, a network of interactions analysis (Gephi 0.9; https://gephi.org) was conducted to evaluate the impact of proposed objectives in connection with COVID-19.

**Table 1.** Participating artists and scientists.

Artists and pieces	Scientists
**Natasha Perdomo**	**Beatriz Arroyo**
www.cowryartstudio.com **Oniric-Binaural landscapes.** In my own way series, 2022 **Recurrencies, Oniric-Binaural landscapes.** In my own way series, 2022	Instituto de Investigación en Recursos Cinegéticos (IREC) (CSIC-UCLM-JCCM), Ronda de Toledo 12, 13005, Ciudad Real, Spain https://orcid.org/0000-0002-4657-6609
**Rafael López-Ramos**	**Julius Nziza**
https://www.lopezramos.info **Breeding lilacs out of the dead land**, 2020 **Pandemic at Pandemonium**, 2020	Gorilla Doctors, P.O. Box 115, Musanze, Rwanda https://orcid.org/0000-0002-9142-0582
**Aimée Joaristi**	**Rita Vaz-Rodrigues**
https://www.aimeejoaristi.com/jardin-del-cielo **Va de pinga.** Heaven’s Garden and Flowers of the Evil series, 2021	SaBio, Instituto de Investigación en Recursos Cinegéticos (IREC) (CSIC-UCLM-JCCM), Ronda de Toledo 12, 13005, Ciudad Real, Spain https://orcid.org/0000-0002-0411-7738
**Aisar Jalil Martínez**	**Mónica Florin-Christensen**
https://www.picassomio.com/aisar-jalil-martinez.html **Exodus** (Exodo), 2020 **Panic** (Pánico), 2020 **The eye and the night** (El ojo y la noche), 2020	Instituto de Patobiología Veterinaria (IPVET), CICVyA, INTA-Castelar, Los Reseros y Nicolas Repetto, s/n, 1686 Hurlingham, Buenos Aires, Argentina. Consejo Nacional de Investigaciones Científicas y Técnicas (CONICET), Buenos Aires C1033AAJ, Argentina. https://orcid.org/0000-0003-0456-3970
**Geandy Pavón**	**Natalie Rudenko**
http://inartwetrustmuseum.org/40days40nights/ **Day 21. Virgin.** Quarantine: 40 Days & 40 Nights series, 2020 **Day 40. Negative Theology: The Black Sun.** Quarantine: 40 Days & 40 Nights series, 2020	Institute of Parasitology, Biology Centre, Czech Academy of Sciences, Branisovska 31, 37005 Ceske Budejovice, Czech Republic
**Sara Stites**	**Juan Mosqueda**
https://www.sarastites.com **Carrier**, 2020 **Wha?**, 2020	Laboratory for Research on Immunology and Vaccines, Faculty of Veterinary Medicine, Autonomus University of Querétaro, Santiago de Querétaro 76010, Mexico https://orcid.org/0000-0001-8892-6390
**José Omar Torres**	**Lorena Mazuecos**
https://www.arteinformado.com/guia/f/jose-omar-torres-176196 **Loneliness** (Soledad), 2020 **Circulating the pandemic II**, 2020	SaBio, Instituto de Investigación en Recursos Cinegéticos (IREC) (CSIC-UCLM-JCCM), Ronda de Toledo 12, 13005, Ciudad Real, Spain https://orcid.org/0000-0002-7897-1304
**Segundo Planes**	**Christopher Binns**
http://www.artnet.com/artists/segundo-planes-herrera/biography Motivated by the COVID-19 pandemic, 2020	IRICA, University of Castilla-La Mancha, Av. Camilo José Cela 1, 13005 Ciudad Real, Spain https://orcid.org/0000-0003-1711-375X

### Collaboration between artists and scientists for research innovation applied to COVID-19

#### Natasha Perdomo. Artist proposal with artworks and comments

**Figure 1.**
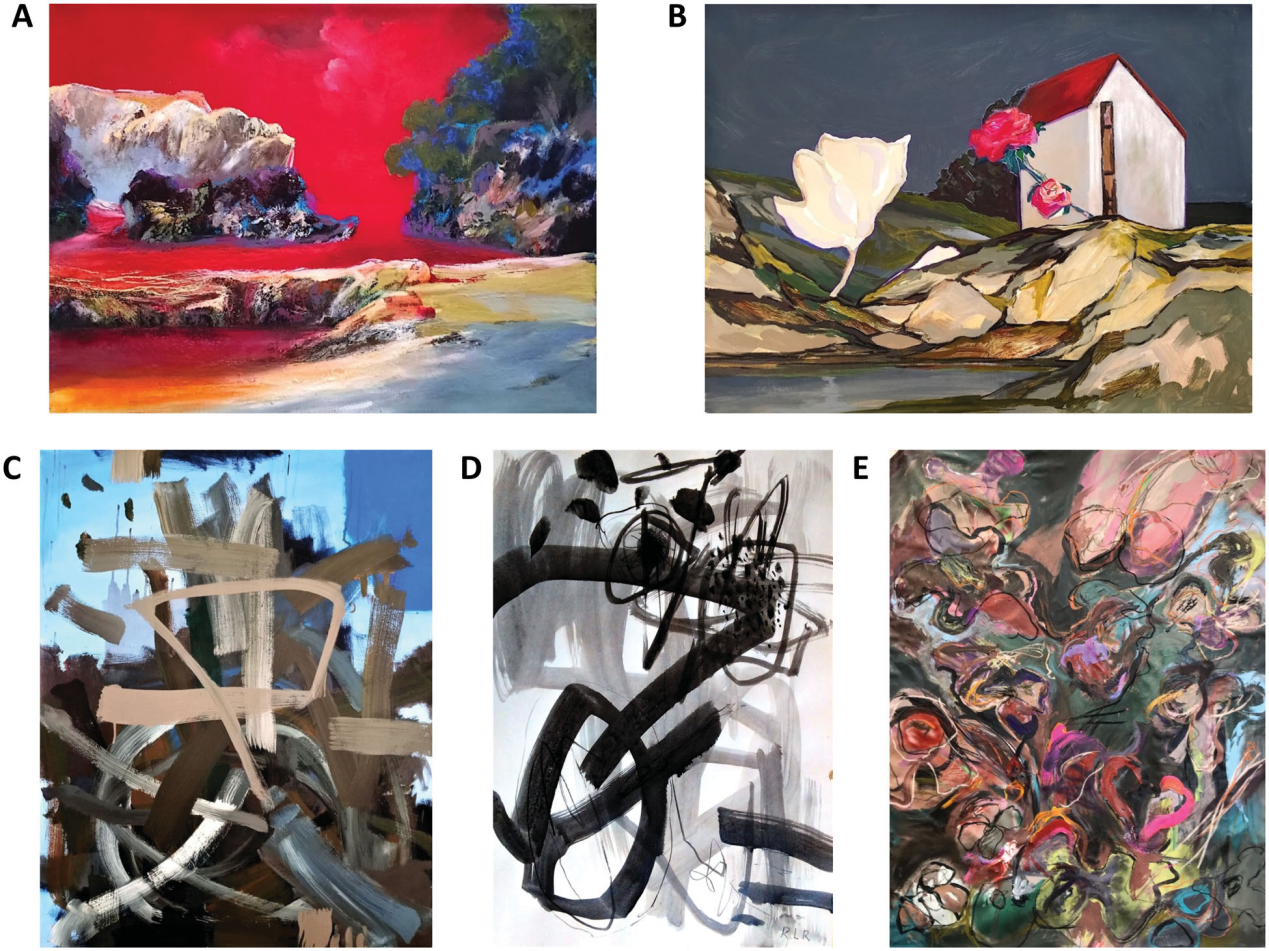
Artworks by N. Perdomo, (A) Oniric-Binaural landscapes, In my own way Series, 2022 (acrylic, oil pastels, soft core pencils on canvas, 30 × 41 cm) and (B) Recurrencies, Oniric-Binaural landscapes, In my own way Series, 2022 (acrylic, oil pastels, soft core pencils on vinyl wallpaper, 30 × 41 cm); R. López-Ramos, (C) Breeding lilacs out of the dead land, 2020 (acrylic on canvas, 99 × 79 cm) and (D) Pandemic at Pandemonium, 2020 (Indian ink on paper, 46 × 30 cm); A. Joaristi, (E) Va de pinga. Heaven’s Garden and Flowers of the Evil series, 2021 (mixed media, 210 × 160 cm).

In the first months of 2020, just when the pandemic began, I decided to take advantage of it to materialize “Off the Grid” (Ongoing Installation Series) (Figure 1(A,B)). Project originated months before when we opened our Cowry Art Studio in a 1920 building. The history of old cities, origins, urban designs, among other details of the environment and coexistence, have led me to attempt an integration of the urban, interior landscape and the natural landscape, which is increasingly scarce in the large cities. Also, in terms of the behaviour of its inhabitants, where modernity prioritizes the man-market relationship, inserting it more and more into a physically and mentally induced being; man is already by himself, a victim of his own confinement where the media and technology represent very aggressive ways that affect that way of thought.

Visual representation has traditionally resorted to a virtual grid in which everything is levelled horizontally and vertically. This rule is rigorously applied both to the exhibition context of the artistic work, and to its printed or virtual reproduction. The paintings in my project tangentially evoke that order, while violating and twisting its rules, to create a flexible matrix that goes beyond its physical and philosophical limits and brings it closer to an energy circuit or a biological network. I reinvent the theme of the landscape, interweaving my painting with the kitsch pattern of the wall covering that supports it. I refer to an industry that gained strength in the United States in 1930, as a symbol of the American dream with its correlate of comfort and prosperity, but also of the control that has been exercised over women as a gender “destined for the environment domestic”, situation reactivated now during the confinement of the pandemic. In an ironic and fanciful staging, my landscapes are intertwined with the flat flowers of the wallpaper, colour that gains saturation and volume, stems that immersed in water seen from the inside or characters that quote tropes from romantic painting, in surreal situations.

All this supported by fine bamboo rods sewn and embroidered in zigzag to the vinyl support, buffalo bone buttons nailed to the wall, inserted into four corner eyelets of each piece of the installation. “Dress up the wall, the house, the Tepee” A metaphorical tribute to the aboriginal culture of North America that has used it materially and spiritually in its everyday crafts, transcending contemporaneity and improving our lives on the planet, through the centuries. https://www.cowryartstudio.com/offthegrid.

During those first months of the pandemic, my mother and younger brother were simultaneously positive for COVID-19 in Cuba. The reality of the epidemic was then much closer and at the same time difficult, without the possibility of being with my family when you needed me most. The creation of my works was the way to express all personal feelings that generated this reality. A daily exercise, a need to express a race against time and operating outside the standards of the art market. New rules and particularities in events and institutions have led the creator to seek their own paths. In my case, be a little freer, without patterns or ties to rules or institutions that govern my life. The world became a chaos, since then it has changed, an evolution that represents a new phase as an artist and as a person. During confinement, my landscapes were reduced to a mixture of views through the window, short walks in the park natural close to home and the inclusion of my dreams. Recurring dreams, altered colours, psychedelic and surreal. Every night I slept listening to binaural sounds, and I moved through those imagined worlds looking for an order. Since that first vision, visually projected between walls and captured on the vinyl panels, I have considered what it represents the word, “home”. Search that goes from the natives of North America with their tipis (practical, nomadic and beautiful in preserving their deep-rooted customs), going through wallpapered house walls, through peculiar kitsch motifs of some local cities or the multicultural incorporation of elements that contribute to the sociocultural environment of their communities. A sequence that has led to parallel works: Oniric-Binaural landscapes, In my own way Series.

Landscapes in small and medium formats were made in acrylic, oil pastels, soft core pencils, oil, charcoal, collage/vinyl, canvas, heavy paper or Canson canvas texture paper. In them the landscapes are already more focussed on her inner world. Humans are a being of clay, their own army on guard, invisible, under wallpaper hanging torn from those walls dreamed I resort to elements of Japanism, just as the impressionists incorporated it into their works, providing a new way of understanding art to transmit feelings and emotions, as a means of expression. Loneliness, personal uncertainty and collective memory of a world– before, during and in the current state of the pandemic – I consider personally that humans have generated in an egocentric state, bringing to the surface the matrix state of survival. Between so much antagonism, individuals have identified their ephemeral condition in the midst of the flaccidity and negligence of the system global economic, political and social Oniric-Binaural landscapes, In my own way Series https://www.cowryartstudio.com/natashaperdomopaintings.

#### Beatriz Arroyo. Scientist view and proposed research inspired by N. Perdomo contribution

The artist’s work emphasizes the need to integrate natural landscapes (and by implication natural ecosystems) with urban and modern life (the “interior landscapes”, “urban designs”, the wallpaper designs representing social constructs for comfort and domestic environments) and breaking the man-market relationships for more “physically and mentally induced beings”. The author also emphasizes that the COVID-19 confinement led to a “world that became a chaos”, and where the interior life (dreams) needs to replace those of outside landscapes, and how humans have generated a word “in an egocentric state”.

From this, I would highlight the need of two lines of research. One, deepening our understanding of the socio-ecological relationships of risks and effects of pandemics, including COVID-19. In other words, addressing e.g. how the rate of infection as well as the likelihood of negative consequences of the illness if infected are modulated by the socio-ecological context of different countries/areas (e.g. overall environmental health, type of economy – local markets vs. decoupled economies – and impacts on the local environment). Secondly, deepening our understanding of the perceptions of society about the relationships between de-coupled environments and the risk of pandemics, including COVID-19, and whether attitudes and discourses about this influence risk-related behaviours.

#### Rafael López-Ramos. Artist proposal with artworks and comments

At the beginning of March 2020 when the quarantine was declared in Miami, we all started spending more time at home than outside, which also allowed me to spend more time at the studio working on my art and further developing the abstract series I started in 2018 (Figure 1(C,D)). This series did not fundamentally change under these new circumstances, but we could say it grew bigger and deeper as a result of me dedicating more hours at the studio.

The new social reality dominated by isolation, risk and fear as a result from the virus, combined with the social and political tension generated by Trump (and Trumpism), made me face artistic creation as a sort of catharsis, while attempting to process all that negative energy and turn it into an object of beauty, able to channel a positive energy that may even bring about some soul healing and spiritual growing.

The two artworks selected are among the first I created during those months, and the painting was the first on canvas from that period of dark clouds; its title quotes a verse from T.S. Eliot monumental poem “The Waste Land”.

#### Julius Nziza. Scientist view and proposed research inspired by R. López-Ramos contribution

The COVID-19 pandemic has brought to human again the reality that the threats of emerging infectious diseases can cause both human live and huge economic losses. With the SARS-CoV-2 being suggested that it is of animal origin, this again brings the importance of more research of animal sources of zoonoses that have more potential to cause pandemics in the future.

I suggest more research on the role of companion animals in the mental/spiritual health of their owners during the pandemic. On the broader level, it is important the surveillance of viruses at the source of potential future pandemics particularly in wildlife at the human-wildlife high risk interfaces.

#### Aimée Joaristi. Artist proposal with artwork and comments

This work is part of a series called Jardín del Cielo y Flores del Mal that I worked on during the pandemic, seeking peace and tranquillity through my garden, and offering a symbolic tribute to so many people who died alone (Figure 1(E)). You can find more of this series, texts, videos and works at: https://www.aimeejoaristi.com/jardin-del-cielo.

#### Rita Vaz-Rodrigues. Scientist view and proposed research inspired by A. Joaristi contribution

Just like this piece, COVID-19 is a devouring illness. Mentally and physically, this diagnosis carries a burden not only to the patient but also to the patient’s family/friends, especially if we are talking about elderly or immunocompromised individuals. All the emotional roller coaster caused by the pandemic can be sharpened if the pain to go through COVID-19 is experienced alone. It takes you in, consumes you and sometimes lets you out. Others go to the intensive care unit (UCI) or, even worse, to the heaven’s garden. Vaccination has come to ameliorate this feeling of impotence that this disease has brought to us. Although all hopes relied on vaccination, it all came crashing down when the ability to stop virus transmission was not as expected. Uncertainty still remains on whether booster vaccination shots are going to be needed periodically over the course of our lives.

In my opinion, it is important to keep searching for new and innovative methods for COVID-19 treatment and prevention. A good proposal is to study orally administrated trained immunity triggering immunostimulants with proven results for other pathologies, such as the heat inactivated *Mycobacterium bovis* or even alpha-Gal biomolecules (solely or combined) to potentially boost this non-specific part of the immune system and help protecting against COVID-19. These types of approaches could particularly focus on risk groups as a form to avoid severe symptomology and ultimately death. Moreover, an innate immune enabler would also be beneficial for other infectious diseases as its nonspecificity works as a cross protective mechanism. The use of an orally administered drug would function as a regular pill prescription, improving logistics and facilitating its intake, even in cases of solitude.

There are still a lot of COVID-19 information that we do not know, particularly concerning disease long-term effects and virus evolution. The instability remains present in our lives as portrayed in this painting series by the artist A. Joaristi.

#### Aisar Jalil Martínez. Artist proposal with artworks and comments

**Figure 2.**
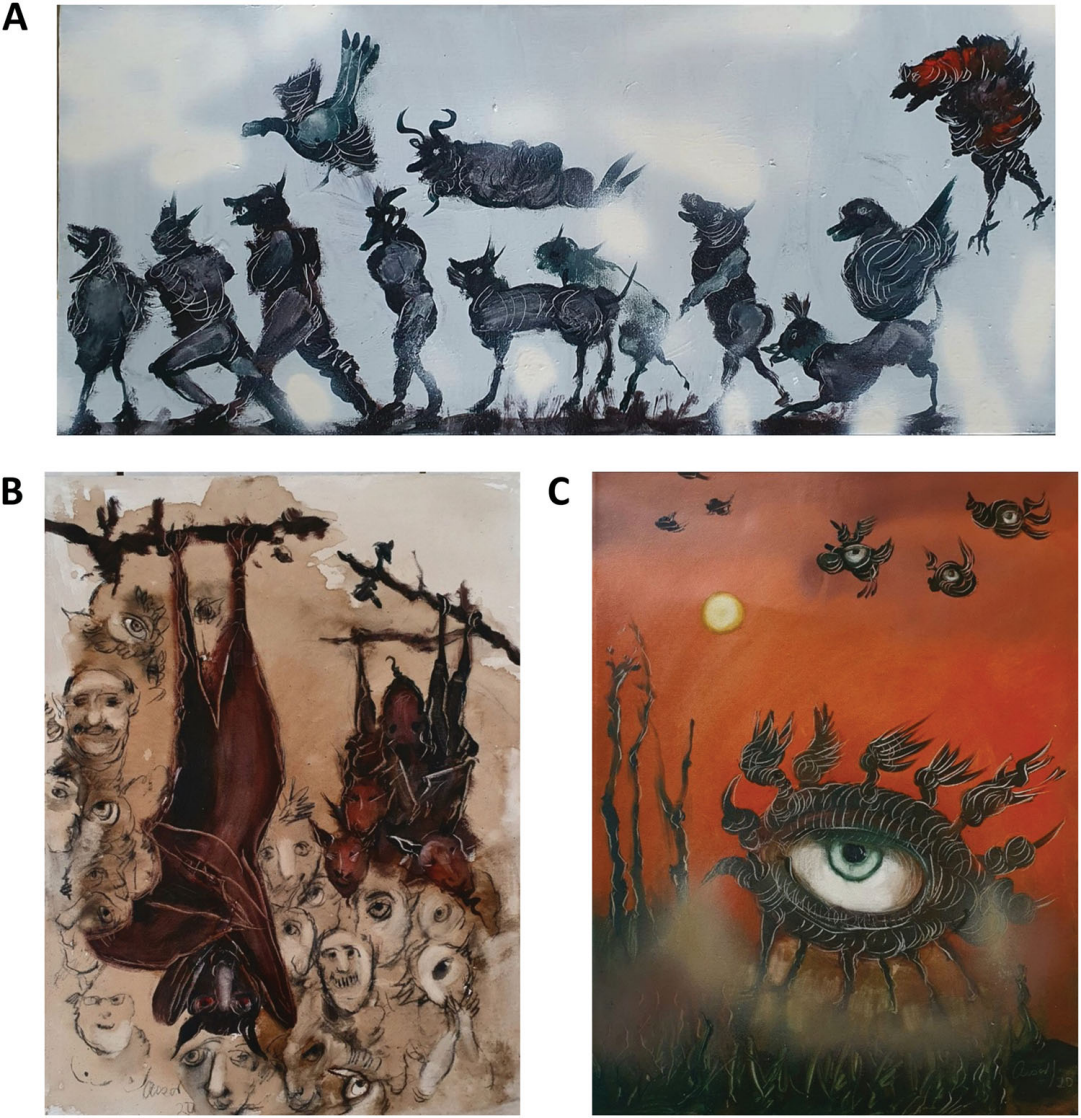
Artworks by A. Jalil Martínez, (A) Exodus, 2020 (oil on canvas, 28 × 63 cm), (B) Panic, 2020 (oil, coffee and pencil on canvas, 63 × 46 cm) and (C) The eye and the night, 2020 (mixed media on canvas, 61 × 46 cm).

These pieces represent the presence of COVID-19 (Figure 2(A–C)). In the piece “The eye and the night” The eye you see is not an eye because you see it; it is an eye because it sees you (inspired by Antonio Machado).

#### Mónica Florin-Christensen. Scientist view and proposed research inspired by A. Jalil martínez contribution

The SARS-CoV-2 is currently the most studied pathogenic virus and a large number of research groups around the world have set up the methodologies needed for its detection. Epidemiological studies on SARS-CoV-2 are represented by the painting “The eye and the night”. Meta-analysis (the big eye) of the local data obtained at different laboratories (small eyes) can throw light on COVID-19 important aspects such as the influence of ethnicity, genetics, climate and other potential risk factors on the impact of the disease. In addition, SARS-CoV-2 epidemiology can be used as a model to understand the dynamics of infectious diseases upon a changing scenario of human and animal spatial distribution. Indeed, policies applied by different countries such as lock-out regulations, banning of work and school attendance, and travel restrictions, have temporarily affected the distribution of people in urban and suburban areas, and their interactions with the environment. Oppositely, the absence of humans in the streets has allowed some wild animal species to re-conquer urban areas. This dynamic could correspond to the artist’s painting “Exodus”. Finally, the painting “Panic” shows a group of people terrified by bats, possibly as prototype of mysterious creatures with unknown powers. From a scientific point of view, this power could correspond to the capacity of bats and other animals to transmit zoonotic diseases, sometimes with devastating consequences on humankind. Also, animals can serve as a “melting pot”, from where recombinant viruses with unpredictable competences can emerge. This painting also reminds us that the original animal reservoir that gave rise to the SARS-CoV-2 pandemics has not been undoubtedly established.

A line of research that could derive from these reflections is to analyse the incidence of zoonotic diseases during and after the COVID-19 pandemic, considering this changing scenario of human-reservoir animal interactions.

#### Geandy Pavón. Artist proposal with artworks and comments

**Figure 3.**
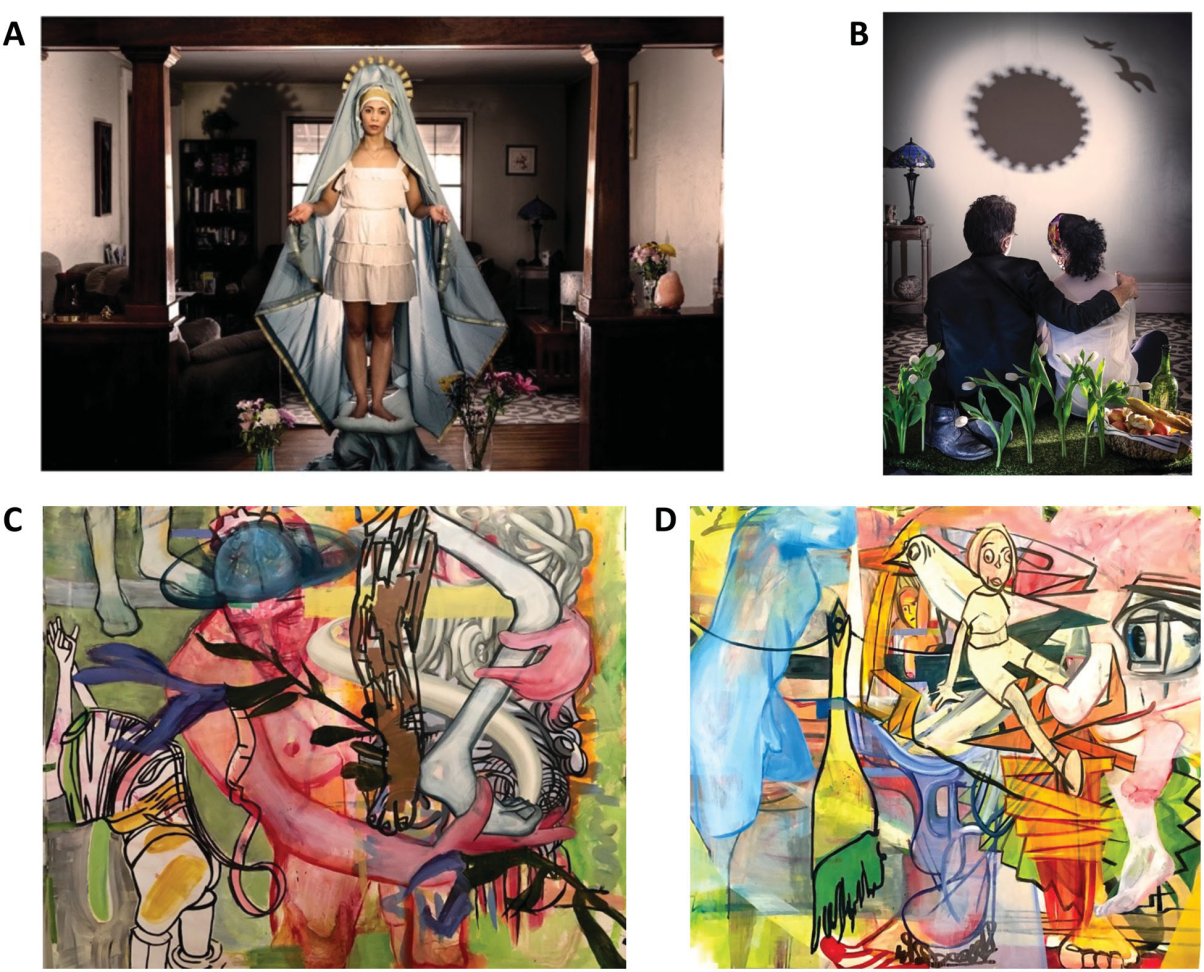
Artworks by G. Pavón, (A) Day 21. Virgin. Quarantine: 40 Days & 40 Nights series, 2020 (photography on Baryta paper like archival pigment prints, 33 × 351 cm) and (B) Day 40. Negative Theology: The Black Sun. Quarantine: 40 Days and 40 Nights series, 2020 (photography on Baryta paper like archival pigment prints, 51 × 33 cm); S. Stites, (C) Carrier, 2020 (oil paint on Yupo paper, 152 × 168 cm) and (D) Wha?, 2020 (oil paint on Yupo paper, 213 × 152 cm).

The series resulted from seclusion during the height of the COVID-19 pandemic (Figure 3(A,B)). According to Lynette M.F. Bosch (http://inartwetrustmuseum.org/40days40nights/), Pavón’s motivation was succinctly expressed by his artist’s statement concerning the pandemic as the catalyst for the series - “The only possible revenge against the pandemic is in the hands of science, in the form of a vaccine, and in art, in the form of catharsis. The need from which I started this series is common to every artist, that vital impulse that leads us to further complicate reality, because art does not explain, but rather complicates reality even more. Making art in seclusion is a challenge: on the one hand you have all the time to perfect an idea, and on the other hand all the material limitations to execute it.” “Quarantine: 40 Days and 40 Nights” and “40 Plus” are the result of Pavón’s desire to make new realities that comment and expand the world in which he finds himself and the circumstances that led to COVID-19 lockdown. It is his way of communicating during his confinement and his record of what he thought about while he avoided the contamination of COVID-19. In creating art during times of plague, Pavón joined a long history of art and cultural change that date from the earliest establishment of towns and cities.

#### Natalie Rudenko. Scientist view and proposed research inspired by G. Pavón contribution

My impression from the artwork: Day 21. Virgin (Figure 3(A)) – congenital transmission of the pathogen. Negative Theology: The Black Sun (Figure 3(B)) – sexual transmission of the pathogen.

The daily life of human population worldwide was affected by COVID-19 pandemic and changed the pattern of social behaviour and acceptance of reality, opened the problems of close inter-human relations inside the family and highlighted the importance of so called “personal space” in society that faced deep restrictions. How prepared was human population for this pandemic? Was it at all? Is it possible to call the human behaviour during restriction time logical or expected? The wave of increasing danger that touched the human mentality highlighted two opposite sides of personal behaviour. One can be called “the Russian roulette”. Jeopardizing with the personal health and wellbeing of society, ignoring the common sense, ignoring restrictions, becoming a “vector” of the virus in its distribution. Another pattern might be called “castaway”. Sensitive personalities accepted restrictions and often “locked themselves down” from the society, from the daily routine, from the danger of being infected, simply developing phobia to reality. Can we predict the behaviour of the virus in two mentioned situations? How much distinct behaviour of human population might affect the lasting of pandemic? Can the “lock down” restrict the virus distribution? May be, distribution by traditionally accepted way. However, each mentioned behaviour pattern still supports the possibility of virus transmission by alternative ways such as sexual transmission or congenital transmission. Either way of behaviour presumably support increase of both. Sexual activities are among the “careless” so as among the “careful” parts of society, with expected baby-boom that must follow-up. And this raises more concerns, i.e. can COVID-19-positive mother transmit the virus to newborn baby? (Day 21. Virgin) or can SARS-CoV-2 be transmitted between sexual partners? (Day 40. The negative theology: The Black Sun). How significant is impact of congenital and sexual transmission of SARS-CoV-2 in the worldwide distribution of COVID-19? The overlook of possible danger does not mean that it does not exist. It deserves to be studied. It is hard to find the black cat in a dark room…especially if no one searches for it.

#### Sara stites. Artist proposal with artworks and comments

The pieces were made during the COVID-19 pandemic and reflect feelings of dislocation and unease (Figure 3(C,D)). The piece “Carrier” shows an actual “carrier”, a man balancing several objects, one a skeletal leg, the other a tree-like form. There is a sense of urgency, as though he is running to safety. The title is a play on the word "carier" that was in the news all the time. It had a somewhat negative connotation although what this man is doing appears to be heroic. On the piece “Wha?”, with an incredible pile up of organic and mechanical form, a figure and a bird, drawn in cartoon like lines, are trying to make sense of the mish mash of experience, memory, art and a somewhat ominous pink sky.

#### Juan Mosqueda. Scientist view and proposed research inspired by S. Stites contribution

Carrier. During the pandemic, millions of people died from COVID-19, causing not only a huge loss of human lifes, but also human resources and loss of mental health. The lack of rapid action to combat the pandemic was largely due to a lack of research on emerging and re-emerging diseases. Investing in research on those pathogens that cause diseases that can become pandemics is necessary. Specialized research groups must collaborate in an interdisciplinary manner with epidemiologists, biotechnologists, immunologists and sociologists to have a more successful approach. But, even more importantly, collaborations between scientists from universities and research centres with companies should be encouraged. In many countries, there is a lack of interaction between research centres and pharmaceutical companies, in such a way that the development of technologies does not reach society on time. Governments must favour interdisciplinary research and academic-industry interaction. Having action groups to fight the next pandemics more quickly and effectively will make pandemics better controlled and fewer lives will be lost.

Wha? The COVID-19 pandemic was a very particular period that changed the lives of many people in many ways. The closure affected scientific research to such an extent that projects were stopped completely or postponed indefinitely. The researchers had to find ways to cope with this situation, including working from home, limited access to laboratories and the difficulty of obtaining materials and reagents. All this, while dealing with issues of home and family. Much of the situation was due to little or poor detection capacity for contagious but asymptomatic people. The development of rapid, but above all, cheap and sensitive pathogen detection methods is necessary. To date, molecular methods, which are expensive and time-consuming, are highly sensitive but require sophisticated equipment and specialized personnel. Immunochromatographic methods are faster, cheaper, do not require equipment or trained personnel, but are not very sensitive, so they only detect the virus in people with clinical signs. Therefore, there is still a lack of detection methods that allow the identification of very low amounts of virus in infected people, but even without clinical signs. These detection methods would allow infected people to be identified before they start transmitting the SARS-CoV-2, facilitating disease control. These methods can be made mandatory in workplaces to ensure low transmission of the disease.

#### José Omar Torres. Artist proposal with artworks and comments

**Figure 4.**
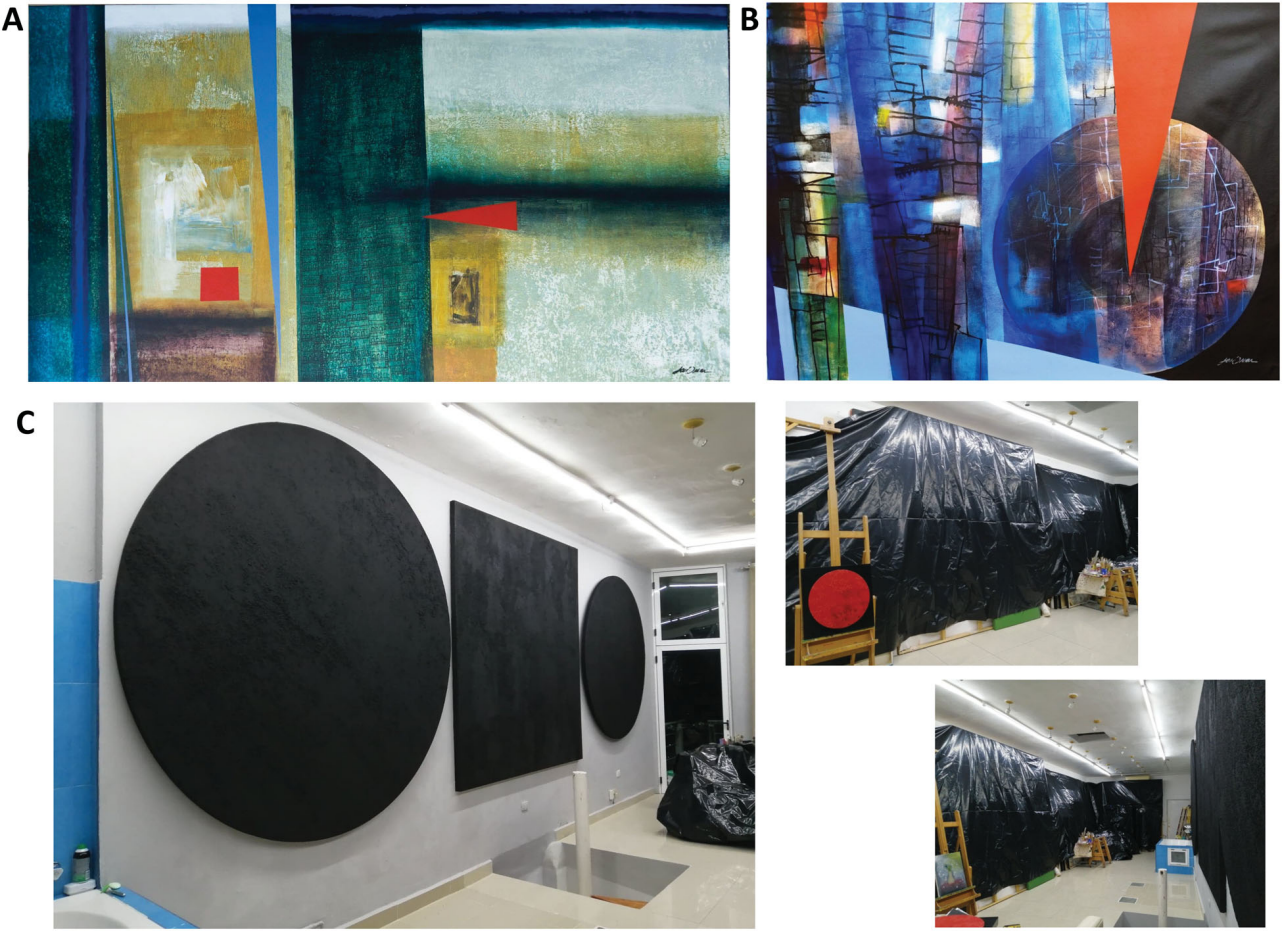
Artworks by J. O. Torres, (A) Loneliness (Soledad), 2020 (acrylic on canvas, 100 × 200 cm) and (B) Circulating the pandemic II (Circulando la pandemia II), 2020 (acrylic on canvas, 95 × 152 cm); S. Planes, (C) Motivated by the COVID-19 pandemic, 2020 (instalation pieces at the artist’s studio, various dimensions).

From "Loneliness" the city is reflected, there is a void, a loneliness in the landscape, the tensions in red locate the problem, the piece is accompanied by a verse of the poet Waldo Leyva "There is so much loneliness in the landscape, so much sea stopped, I travelled so much without going or coming back" In the two pieces of “Circulating the pandemic”, circles of contagion are located, drug work begins, a future is glimpsed, from the vaccines the colour changes in the way of working and works confined but science gives us hope, that hope became a reality with vaccines (Figure 4(A,B)).

#### Lorena Mazuecos. Scientist view and proposed research inspired by J. O. Torres contribution

Loneliness reflects to me to a hospital centre in many characteristics. First, I can feel when observe the picture the particular feeling that you could sense in March 2020 in a hospital, where patients had to be alone in their rooms with no company at all, except doctors and nurses. A combination between fear, loneliness, unknowledge and, to a lesser extent, guilt. Next, the paths and lines together look like empty halls and long dark corridors from a different perspective, with no one in, where a red alert was persistent in the environment, represented by the two red points. Colours and textures are not clearly defined, being in some places only strokes and brushstrokes, except the two red symbols brightly defined that break with the rest of the pattern of the picture. That may represent the fear and the virus, that broke us in many senses at that moment. You can observe that only two compartments (rooms) are well-defined, that could reflect different areas of the hospital. That lack of accuracy or precision in both painting and colours might represent the hesitance that existed specially in hospital centres in the beginning of the pandemic, in which the future was uncertain and the data were not encouraging. In some places, a little picture or a background seems to appear, but with no-strong enough, same metaphoric movement than different pandemic waves one behind another in 2020. When the data encouraged optimism, another new wave of infections did not leave this feeling being awaked. Finally, the title “loneliness” accompanies the situation I described before in a totally personal manner, being the main feeling in patients and health professionals.

Circulating the pandemic II represents for me the whole world progress against the pandemic. Colours, paths and textures are totally different from the previous illustration (Loneliness). The red symbol is still present in an alert manner, that persist representing the biological threat over the world. However, the colours and figures that accompanied the target of this symbol are different. This target (main circle on the illustration) may represent the world, which is facing this biological agent almost at the same time. Indeed, the red symbol intends to break the stability of the figure, as SARS-CoV-2 did in all fields. Nevertheless, this circular figure (target) contains inside a pattern of lines like a puzzle that expands throughout the entire illustration (inside the circle and outside), awakening more intense colours and more define settings. We can observe green, blue, orange and pink, that are described as warm colour that represent different positive feelings like warmth, closeness, vitality, enthusiasm, dynamisms and joy. The author may have chosen these combinations of bright colours and lines due to the perspective that we have now on the pandemic.

At the beginning, the whole world was shocked by COVID-19, but the different fields (science, education, politics, economy) have to work together in a multidisciplinary manner to deal and manage the crisis to achieve a common goal: stabilization.

#### Segundo Planes. Artist proposal with artworks and comments

Motivated by the COVID-19 pandemic, I covered the entire studio in black and did some works without images (Figure 4(C)).

#### Christopher Binns. Scientist view and proposed research inspired by S. Planes contribution

This work for me inspires the thought that to tackle a complex multi-faceted societal problem like the COVID-19 pandemic, whose lack of understanding is inspired by covering the studio in black, it is important to study the interaction between disparate areas of knowledge (inspired by the touching squares and circles). Some of which are not well understood (inspired by the featureless black), for example, the effect of societal changes on human-animal interactions, and the effect of environmental pollution on the uptake of the virus.

The collaboration between artists and scientists resulted in scientific objectives (artist-scientist) with ongoing investigations and related publications.

#### Objective 1 (A. Joaristi – R. Vaz-Rodrigues)

To study orally administrated trained immunity triggering immunostimulants with proven results for other pathologies, such as the heat inactivated *Mycobacterium bovis* or alpha-Gal biomolecules (solely or combined) to potentially boost non-pathogen specific components of the immune system and contribute to protection against COVID-19. The role of biomolecules and processes in trained immunity and the evolution of host-pathogen interactions has been proposed with some evidence for the study of disease aetiology and the identification of new therapeutic interventions for the control of COVID-19 and other infectious diseases [22–25]. Ongoing investigations address the characterization of immune response and nutritional status biomarkers and antibody response to alpha-Gal in COVID-19 patients before and after vaccination. The results will contribute to a better understanding of vaccine protective mechanisms and the development of nutritional interventions with possible impact on boosting protective response to vaccination.

#### Objective 2 (S. Planes – C. Binns)

To study the effect of social changes on human-animal interactions, and the effect of environmental pollution on the uptake of the SARS-CoV-2. Host, virus and environmental-derived factors drive the COVID-19 pandemic [26,27]. Human–human, human–animal and animal–animal interactions in both urban and rural settings may drive the appearance of new virus variants with impact on human and animal health [28,29]. Therefore, a One Health multidisciplinary approach is necessary for a more effective control of COVID-19 and prevention of future pandemics [30]. An ongoing investigation addresses the question on how long SARS-CoV-2 survives on the surface of particulate matter (PM) from different origin to evaluate the relationship between fuel and atmospheric pollution and virus transmission risk. The results provide additional evidence to link the impact of fuel PM pollutants on host immunity with virus transmission and prevalence of COVID-19 and potentially other infectious diseases [31,32].

#### Objective 3 (R. López-Ramos – J. Nziza)

To study the role of ectoparasite vectors in the appearance of SARS-CoV-2 variants and virus transmission. The study of human-animal interactions for companion animals and wildlife and their role in both human and animal health was addressed in Objective 2. The positive psychosocial effect of companion animals during COVID-19 has been partially characterized [33]. However, virus surveillance in domestic animals and wildlife may serve to monitor the appearance of new virus variants and investigate further the role of ectoparasite vectors in the biological (active) and/or mechanical (passive) transmission of the virus [30,34,35]. Currently, we are characterizing the *in vitro* interaction of SARS-CoV-2 Spike (S) protein receptor-binding domain (RBD) with membrane-associated carboxypeptidase angiotensin-converting enzyme 2 (ACE2) receptor from different ectoparasite species. The results will support or refute the RBD-ACE2 interactions predicted *in silico* [35].

#### Objective 4 (J.O. Torres – L. Mazuecos)

To monitor coronavirus circulation in rural communities. A method was developed using Dry-Sponges (3 M-España, Madrid, Spain) for sampling of high-use surfaces and clothes in homes and public service sites [36]. The collaboration with citizens, public health service, administration and business owners allowed the collection and analysis of samples over various months to evaluate the circulation of the SARS-CoV-2 and correlation with diagnosed cases. The results support the collaboration between different sectors to contribute to virus surveillance and reduce risks of contagion.

#### Objective 5 (A. Jalil martínez – M. Florin-Christensen)

To characterise the incidence of zoonotic diseases during and after the COVID-19 pandemic in relation to modifications in the interactions between humans and reservoir animal species. Humans have always been exposed to zoonotic diseases [37]. Although related to Objective 2, the study of the incidence of zoonotic diseases may be modified by the COVID-19 pandemic. For example, although travel restrictions may reduce human-animal interactions, the contact between wildlife and livestock may increase thus increasing the risk of animal-to-animal pathogen transmission and the appearance of new zoonotic diseases [28]. The implementation of new tools for the identification of pathogenic microorganisms in reservoir animal species is an ongoing project through environmental sampling. The results will contribute to reducing the risks associated with new potential zoonotic pathogens.

#### Objective 6 (G. Pavón – N. Rudenko)

To evaluate the risks associated with sexual or congenital transmission of SARS-CoV-2. Current evidence supports coronavirus transmission by semen but not through vaginal fluids [38]. Congenital transmission of SARS-CoV-2 has been documented with clinical-pathological features [39,40]. Therefore, SARS-CoV-2 sexual and transplacental transmission may be considered in epidemiological studies. Special attention should be given to co-transmission with other pathogens with increasing incidence worldwide. For example, open for discussion is the possible sexual or congenital transmission of *Borrelia burgdorferi* sensu lato (s.l.) complex, the causative agent of tick-borne Lyme borreliosis [41]. The results will contribute to the characterization of pathogen–pathogen-host interactions with clinic-epidemiological implications.

#### Objective 7 (S. Stites – J. Mosqueda)

To develop SARS-CoV-2 detection methods for the easy and rapid detection of very low virus amounts in infected but asymptomatic individuals. Current easy-to-handle rapid antigen tests for SARS-CoV-2 detection are not very sensitive [42]. These detection methods would allow the identification of infected people before they start transmitting the virus to facilitate disease control, particularly in areas with poor infrastructure. Promoting interdisciplinary research and academic-industry collaboration would facilitate this process.

#### Objective 8 (N. Perdomo – B. Arroyo)

To better understand the perceptions of society about the socio-ecological relationships between decoupled environments and the risks and effects of pandemics such as COVID-19. Addressing how local socio-ecological factors such as environmental health, type of economy (local markets vs. de-coupled economies) and impacts on the local environment affect virus infection rate and disease consequences would contribute to mitigating the impact on society of COVID-19 and future pandemics.

### Impact of the proposed objectives

**Figure 5.**
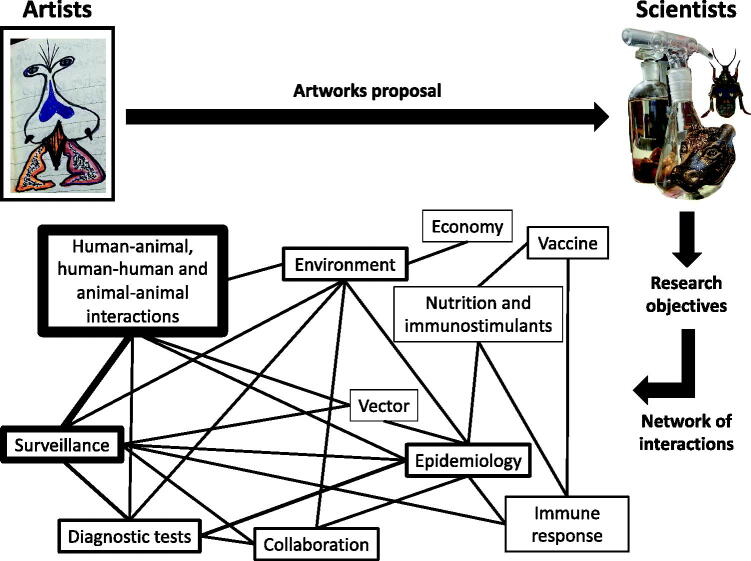
Workflow of artist–scientist interactions and proposed research objectives. Network interactions of research objectives proposed from the collaboration between artists and scientists to advance the study of the challenges posed by the COVID-19 pandemic. Enrichment of terms and interactions correlates with line width. Images are courtesy of the author.

The research objectives proposed from the collaboration between artists and scientists advance the study of the challenges posed by the COVID-19 pandemic with potential applications to other infectious diseases (Figure 5). The characterization of the immune response in in vaccinated and non-vaccinated individuals SARS-CoV-2 PCR-negative and PCR-positive with different COVID-19 symptomatology is key to advance in vaccine design and implementation as well as for disease epidemiology and surveillance. The relationship between nutritional status and response to vaccination is a key component of the possible differences in the individual immunity elicited by vaccines and thus using immunostimulants may contribute to protection against COVID-19. The study of human-animal interactions is important to monitor the appearance of new coronavirus variants and their impact on both human and animal health. How pollution impacts on human and animal health with respect to infectious and non-infectious diseases is relevant to further support the need for interventions to limit global environmental change. The characterization of the role of ectoparasite vectors in the biological and/or mechanical transmission of the coronavirus may be relevant for the appearance of new variants and their transmission cycle. The collaboration between different sectors facilitates coronavirus surveillance to reduce risks of contagion with emphasis on isolated rural communities. The implementation of effective protocols for environmental sampling will contribute to the identification of new potential zoonotic pathogens. Epidemiological studies considering possible sexual and congenital SARS-CoV-2 co-transmission with other pathogens are important to reduce disease-associated risks and implement effective control measures. Early detection of SARS-CoV-2 in different settings using highly sensitive easy-to-handle rapid antigen tests will facilitate disease surveillance and control. Promoting the collaboration between research institutions and pharmaceutical industry in different countries will facilitate preparedness to face future pandemics and reduce its impact on society. Decoupling will allow economic growth without corresponding increases in environmental pressure for a better prepared society to face current and future pandemics.

The network of interactions analysis identified human–animal, human–human and animal–animal interactions followed by surveillance, test, environment and epidemiology as the proposed tasks with highest impact on COVID-19 (Figure 5). The most prominent interactions were between human–animal, human–human and animal–animal interactions and surveillance followed by surveillance-diagnostic test and diagnostic test-epidemiology interactions (Figure 5).

## Conclusions

The proposed methodological approach with the collaboration between artists and scientists resulted in novel and challenging research objectives to address scientific questions with social and economic implications. The main limitation of this study is that artists and scientists did not contact each other, and only the author has the possibility to interact with them. As previously proposed [7], a better approach is that visual and musical artists interact with scientists involved in different research projects to get access to diverse views of the same challenge, question or objective. The different artistic communications should be presented to scientists for an inspiration in their research to describe new hypotheses and directions inspired by these pieces. The participation of students interested in the project is also relevant. Multidisciplinary interactions between scientists with different scholarship and areas of expertise like in this study and visual artists and musicians potentiate the potential outcomes of the project.

## Data Availability

Data sharing is not applicable to this article as no new data were created or analysed in this study.
